# Congenital Syphilis: A Case Report Presenting a Rare Clinical Manifestation in Two-Month-Old Newborn in Bahrain

**DOI:** 10.7759/cureus.56005

**Published:** 2024-03-12

**Authors:** Zaynab B Maqwar, Ammar S Alalawi

**Affiliations:** 1 Pediatric Medicine, Al-Amiri Hospital, Kuwait City, KWT; 2 Pediatric Medicine, Salmaniya Medical Complex, Manama, BHR

**Keywords:** hepatosplenomegaly, failure to thrive, maculopapular rash, treponema pallidum, congenital syphilis

## Abstract

Congenital syphilis, caused by the Gram-negative obligate bacterium *Treponema pallidum*, can manifest as early- or late-onset infection, typically exhibiting classic symptoms such as a maculopapular rash, failure to thrive, and hepatosplenomegaly. This case report presents rare clinical manifestations of congenital syphilis not typically observed during early onset infection in a newborn in Bahrain. Additionally, it details the physical findings and investigations conducted to diagnose the disease.

## Introduction

Syphilis is a sexually transmitted disease caused by a Gram-negative obligate bacterium spirochete called *Treponema pallidum*, which can be classified as an acquired infection, when *T. pallidum* enters the body through various means, including sexual contact, contaminated needles, or direct contact with the skin lesion of an infected person. It can also be classified as a congenital infection, as *T. pallidum* can be transmitted from the mother to the fetus transplacentally or during labor when traversing the vaginal canal. This may result in fetal loss, preterm delivery, and stillbirth, or affect the newborn resulting in congenital syphilis [1].

Congenital syphilis is associated with significant morbidity and mortality [2]. Despite an increased incidence in recent decades worldwide, including in developed countries, early detection and treatment remain crucial [2].

Based on the onset of the clinical symptoms, congenital syphilis can be classified as early or late. Early congenital syphilis is an infection in which clinical symptoms start to appear within the first 2 years but most commonly within the first 3 months after delivery [1]. Approximately 40% of early congenital syphilis are stillborn in untreated pregnancies [1]. A baby with early congenital syphilis can either be asymptomatic, or present with nasal congestion, pneumonia, hepatosplenomegaly, hyperbilirubinemia, cholestasis, maculopapular rash, and lymphadenopathy. Moreover, early congenital syphilis can affect the central nervous system and cause meningitis or seizures [3]. Osteochondritis, periostitis, and Parrot pseudoparalysis are the skeletal manifestations of the disease [4]. In 1871, Jules Marie was the first to describe the pseudoparalysis of Parrot by decreased range of movement of the joints, mainly in the upper limb, due to painful periostitis that can be detected by radiographs in 95% of the cases [4]. Here, we report the first child in Bahrain who presented with Parrot pseudoparalysis of the right upper arm, hepatosplenomegaly, abdominal destination, and anemia and was diagnosed with congenital syphilis.

## Case presentation

A 2-month-old Bahraini baby girl was delivered at term via normal vaginal delivery without any complications. Her APGAR score was 9/10 at 1 minute. She weighed 2.8 kg at birth, with a head circumference of 33.4 cm and an abdominal circumference of 35 cm. Throughout the neonatal period, she remained asymptomatic and did not develop jaundice; her skin color remained normal. Physical examinations revealed no abnormalities across all systems.

During prenatal care, the mother, gravida 2 and parity 2, received routine prenatal care, including surveillance and screening for TORCH (toxoplasmosis, rubella, cytomegalovirus, herpes simplex, and HIV) and anemia, with multiple visits to an obstetrician. During these visits, she was found to have iron deficiency - anemia during pregnancy, but it was controlled after giving her 30 mg of ferrous iron from week 13.

The baby received her first vaccination at birth and was scheduled for a two-month vaccination at a healthcare center. However, due to inadequate weight gain, which resulted in her weight being 3.7 kg, the vaccination was deferred, and she was referred to Salmaniya Medical Complex (SMC) Hospital in Bahrain for further evaluation. The infant's feeding pattern exhibited suboptimal intake, necessitating exclusive reliance on milk formula. Notably, there were no reported instances of vomiting, diarrhea, or irritability. The mother attributed concerns regarding inadequate weight gain and abdominal distension to breastfeeding, prompting the transition to formula feeding.

**Figure 1:**
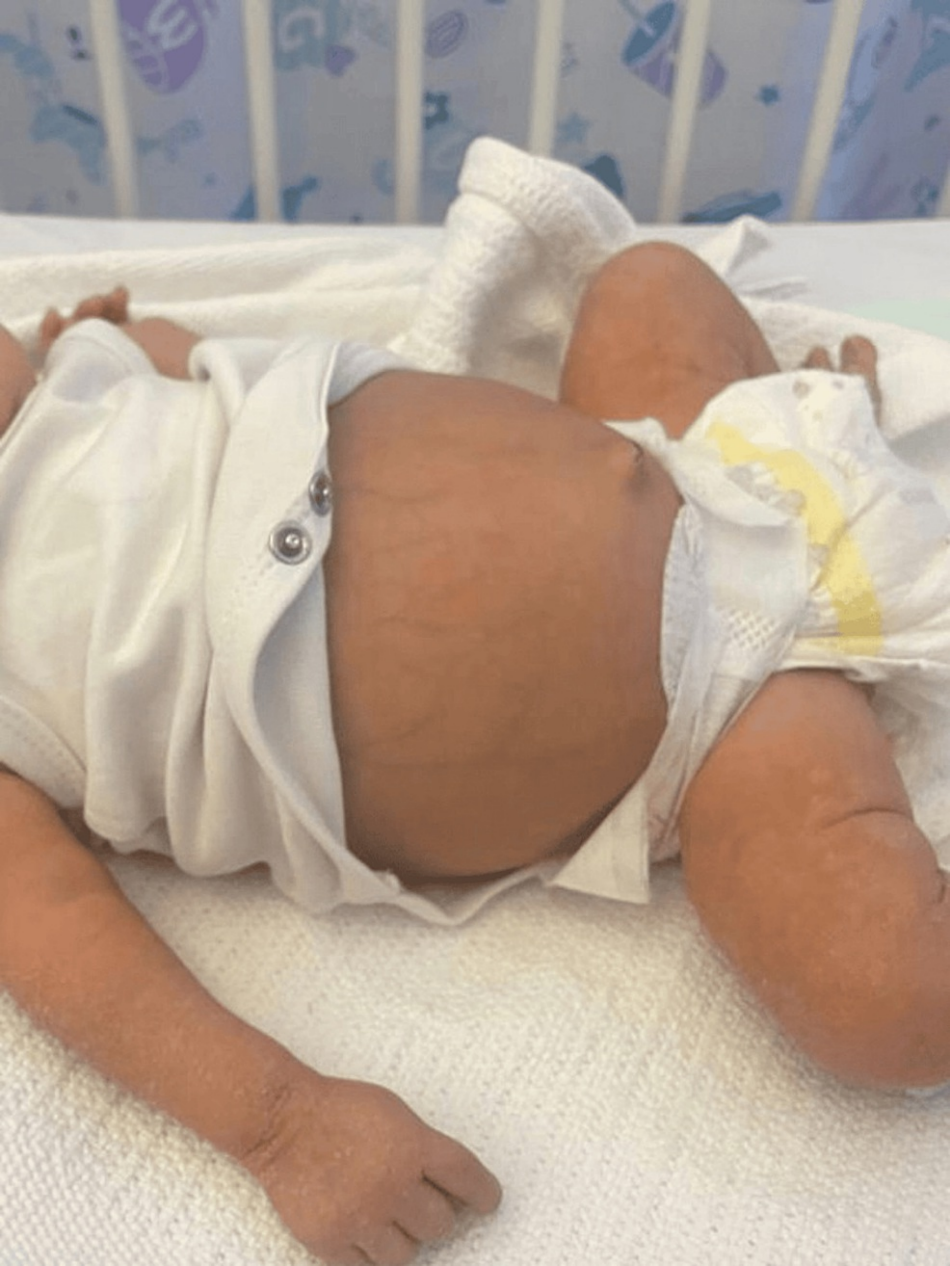
Early congenital syphilis newborn with abdominal destination and dilated vein.

Upon physical examination, in general inspection, the baby was looking pale, dehydrated, and hypoactive. In the central nervous system examination, the baby’s right arm was paralyzed and swollen with excessive crying on moving the right shoulder and a flat anterior fontanelle with a dilated vein of the scalp. These findings were not observed by the mother. In abdominal emanation, the baby’s abdomen was distended with a visible dilated vein (Figure 1), and hepatomegaly. In the cardiovascular examination, normal heart sounds S1 and S2, and no click or gallop systolic ejection murmur were observed.

The laboratory results displayed elevated white blood cell count, inflammatory markers, and reticulocyte levels, and abnormal liver function test findings. Furthermore, TORCH testing indicated a positive result for cytomegalovirus (CMV), and the *T. pallidum* hemagglutination assay was reactive, as illustrated in Table 1. 

**Table 1:** Lab results ESR: erythrocyte sedimentation rate; anti-HBS: hepatitis B surface antibody; VDRL: Venereal Disease Research Laboratory; C+S: culture and sensitivity

	Lab Results	Unite	Reference Range
White blood cell count	14.93	x10^9^	3.6–9.6
Hemoglobin	7	x10^12^	12–14.5
Platelets	299	x10^9^	150–400
Reticulocyte	6.8	%	0.5–1.5
Urea	1.8	mmol/L	3.2–8.2
Creatinine	19	μmol/L	18–35
Albumin	29	g/L	39–54
Alkaline phosphate	257	U/L	150–420
Alanine aminotransferase	63	U/L	≤33
G-glutamyl transferase	69	U/L	≤38
ESR	135	Mm/hour	≤20
C-reactive protein	83.65	mg/L	0–3
VDRL-rapid plasma reagin-syphilis screen	Negative		
*Treponema pallidum* hemagglutination	Positive		
Anti-cytomegalovirus IgG	12.4		
Anti-HBS	1	mIU/mL	Negative
Ammonia	17.87	μ/dL	15–45
Homocysteine	11.11	Micmol/L	<15
Vitamin B12	321	Pg/mL	160–950
HIV DNA PCR	Negative		
Blood peripheral C+S after 48 hours	Culture sterile		
Urine midstream C+S	Culture Sterile		

**Figure 2:**
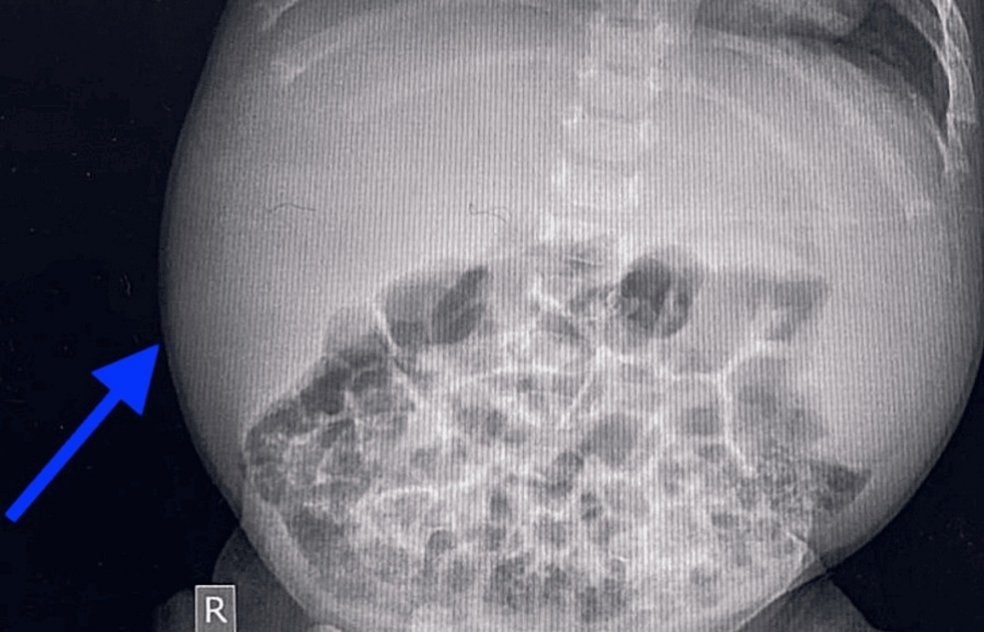
Abdominal X-ray scan of a two-month-old baby girl presented with abdominal destination, hepatosplenomegaly, and diagnosed with congenital syphilis.

**Figure 3:**
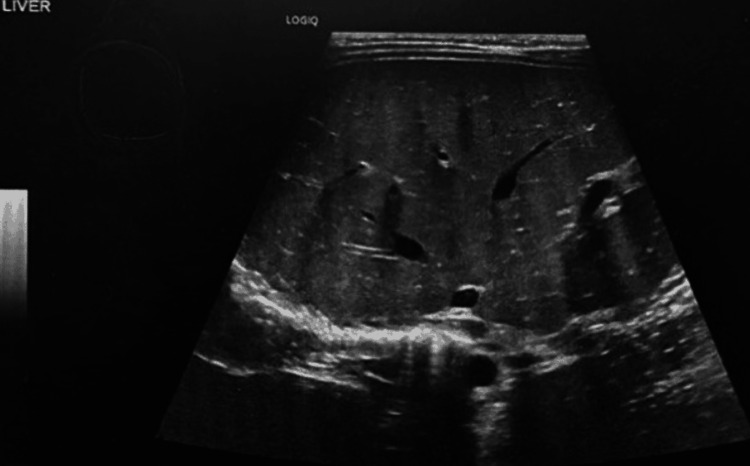
Abdominal U/S scan shows hepatosplenomegaly and mild ascites.

**Figure 4:**
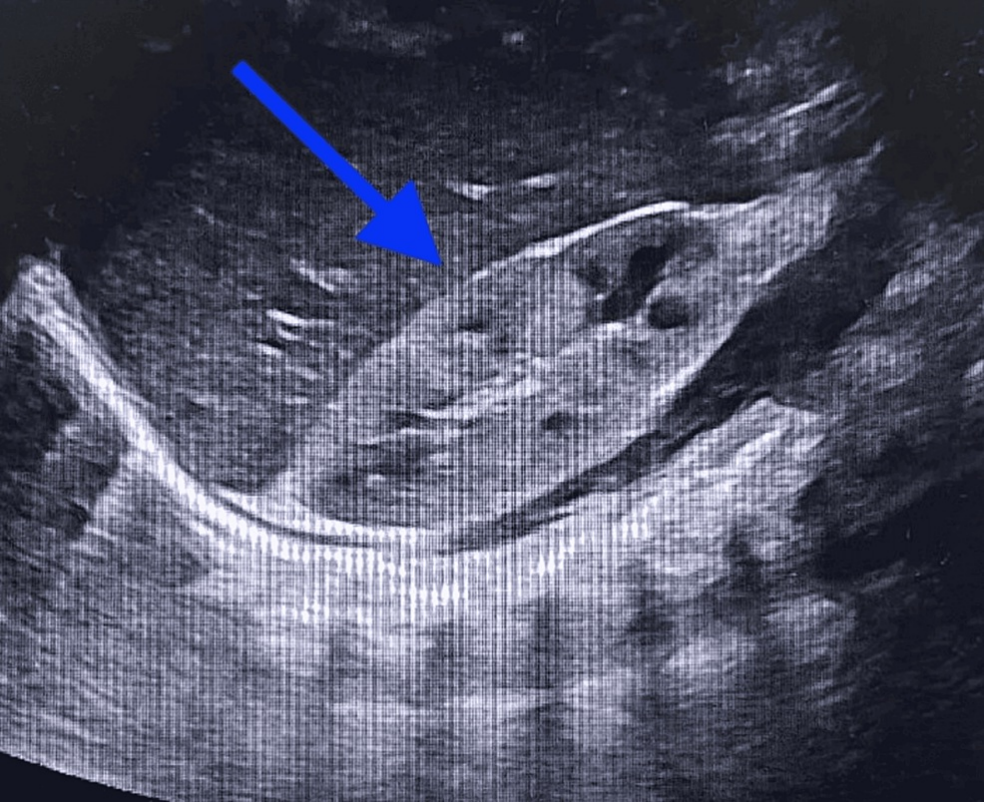
Abdominal U/S scan shows hepatosplenomegaly and mild ascites.

**Figure 5:**
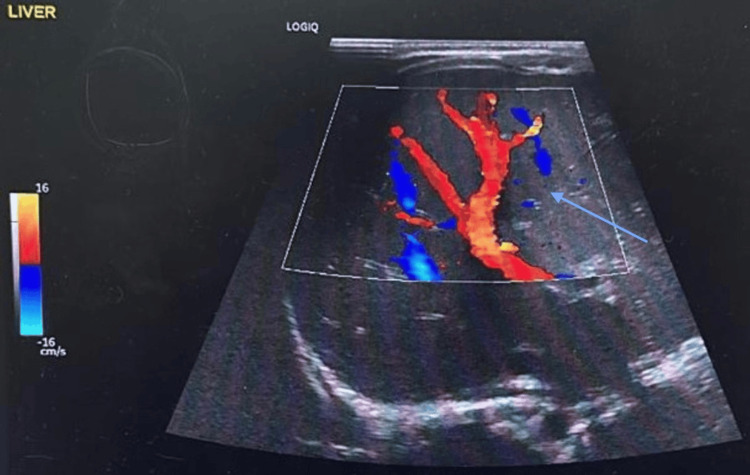
Abdominal U/S scan shows hepatosplenomegaly and mild ascites.

Abdominal X-ray scan showed a very distended abdomen, possibly hepatosplenomegaly or ascites (Figure 2). Hence, an abdominal ultrasound was ordered and confirmed hepatosplenomegaly and mild ascites (Figures 3-5).

**Figure 6:**
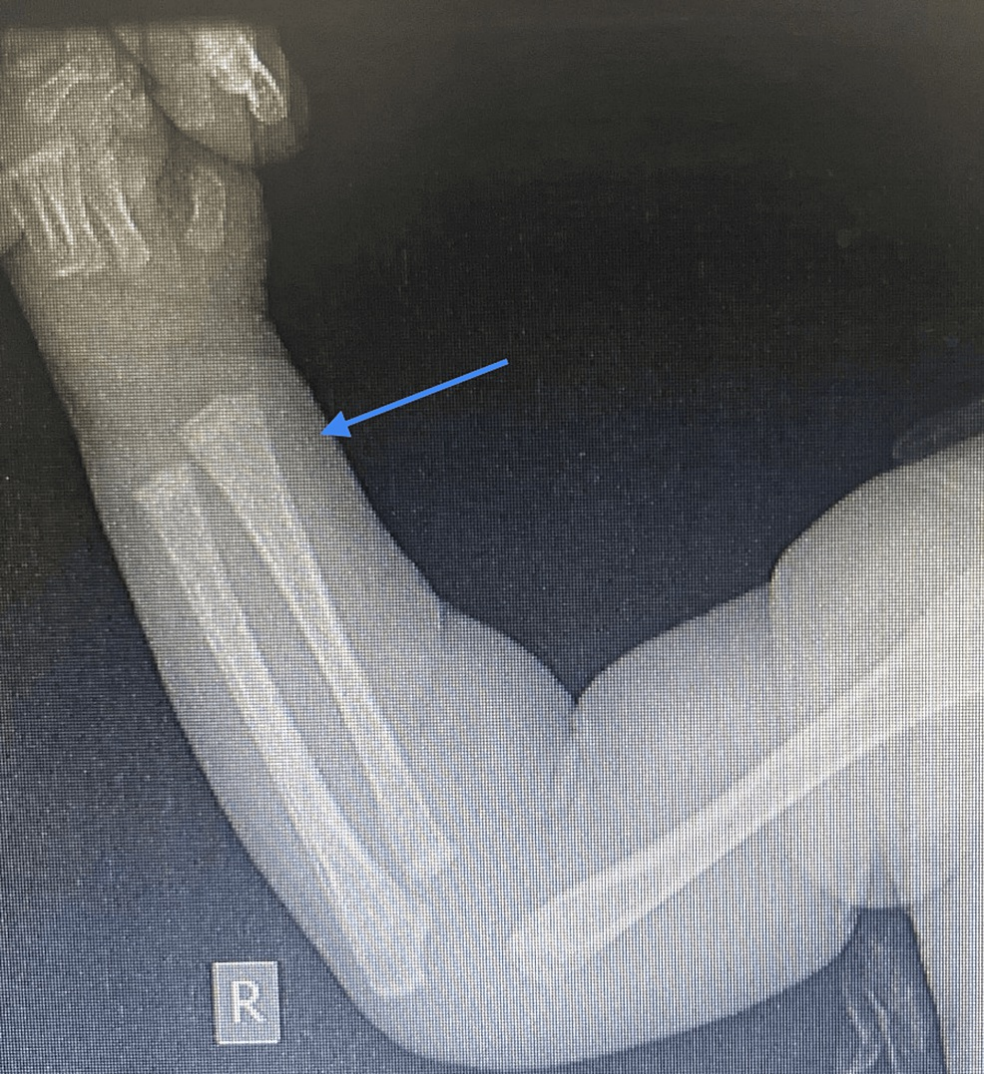
X-ray scan of periosteal reaction in the right radius.

**Figure 7:**
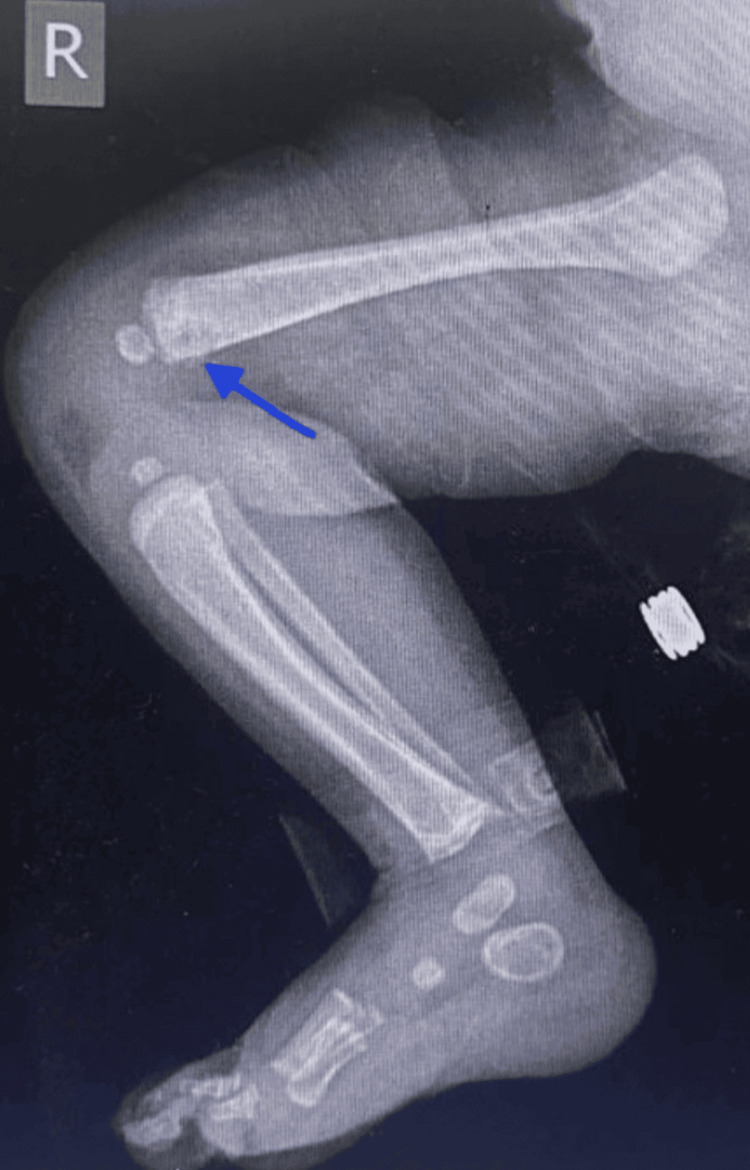
X-ray scan shows area of destruction in the distal shaft of the right femur.

**Figure 8:**
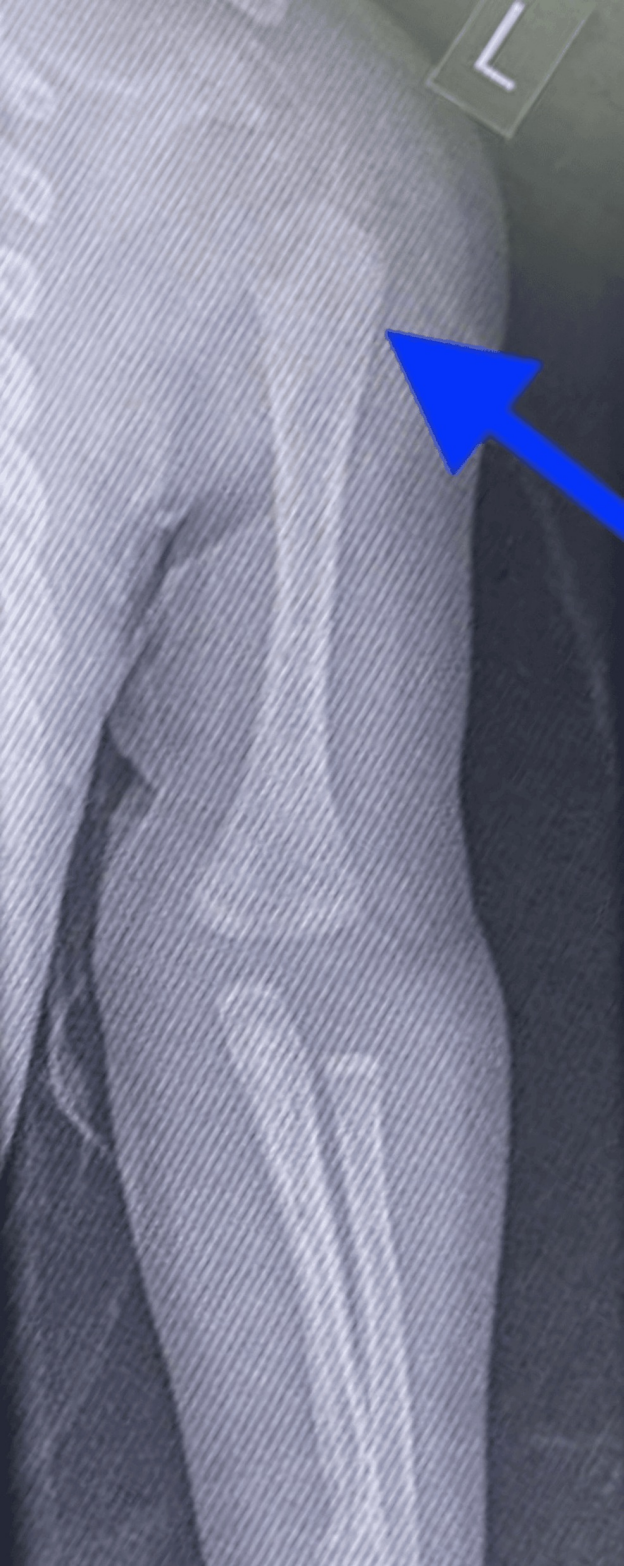
X-ray scan shows area of destruction in the proximal shaft of the left humerus.

**Figure 9:**
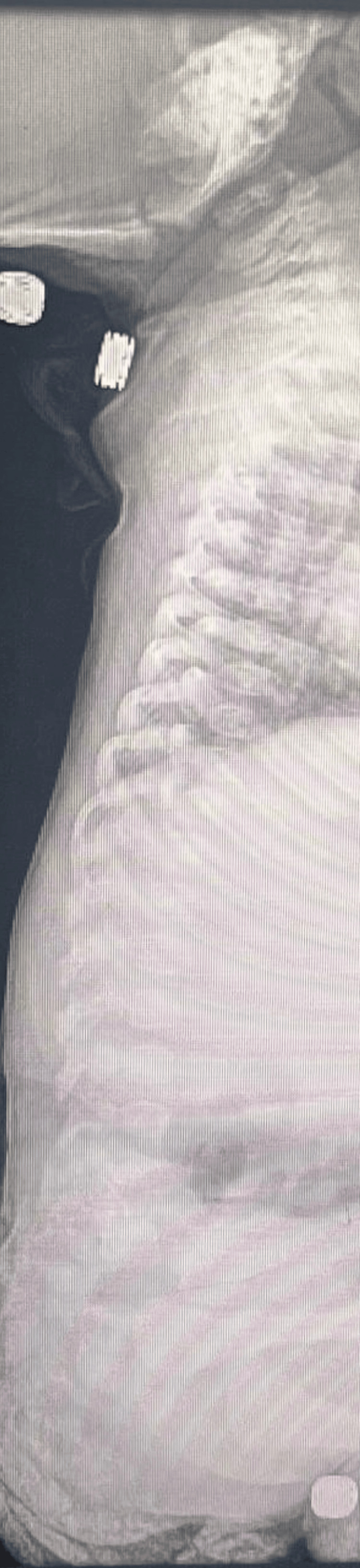
Skeletal survey: The thoracic cage, the spine, and the pelvic bones appear normal.

An X-ray of the swelling in the right arm showed a periosteal reaction in the right radius with small cystic lesions in the proximal humerus and abnormal bone quality (Figure 6). A skeletal survey showed that the thoracic cage, spine, and pelvic bones appeared normal (Figure 9). However, areas of destruction were observed in the proximal shaft of the left humerus (Figure 8) and the distal shaft of the right femur (Figure 7). Despite these findings, the baby's left arm and right leg appeared intact and remained active during the physical examination. The U/S of the right arm revealed suspected osteomyelitis.

**Figure 10:**
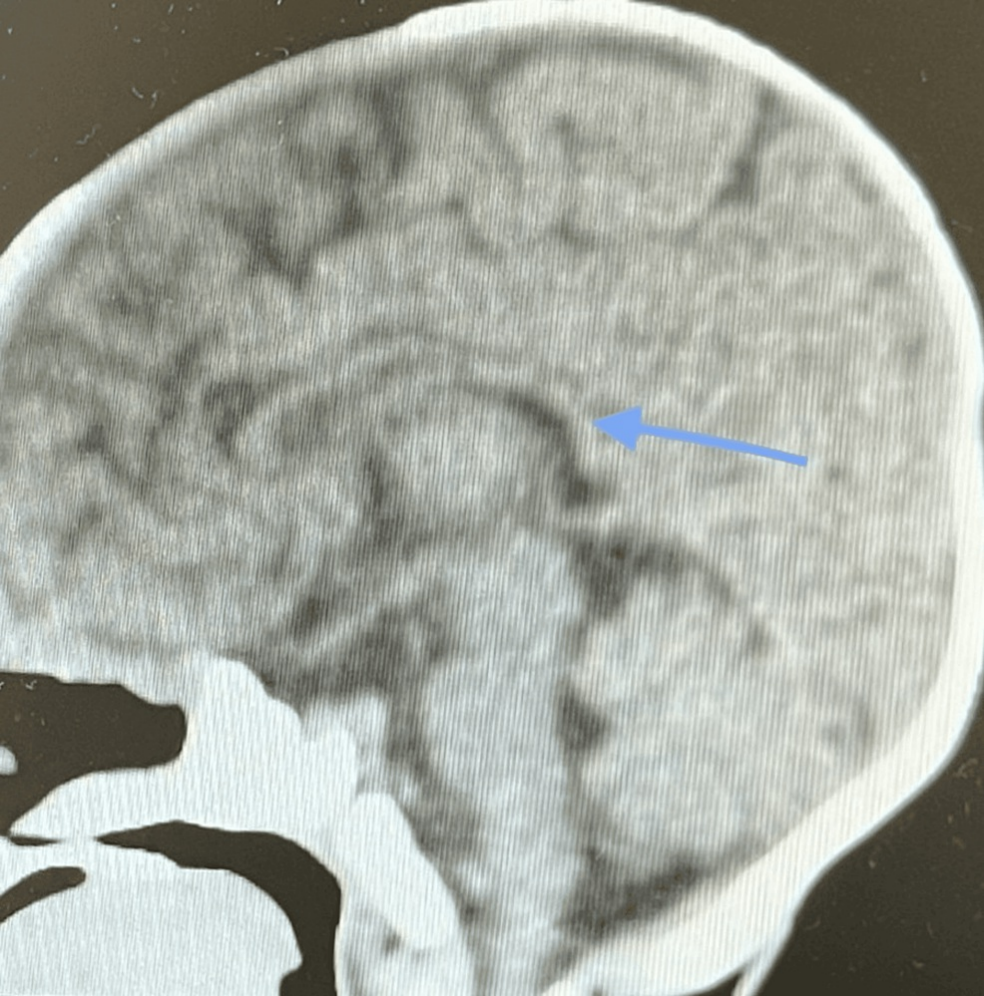
Non-contrast CT brain scan: thin corpus callosum.

**Figure 11:**
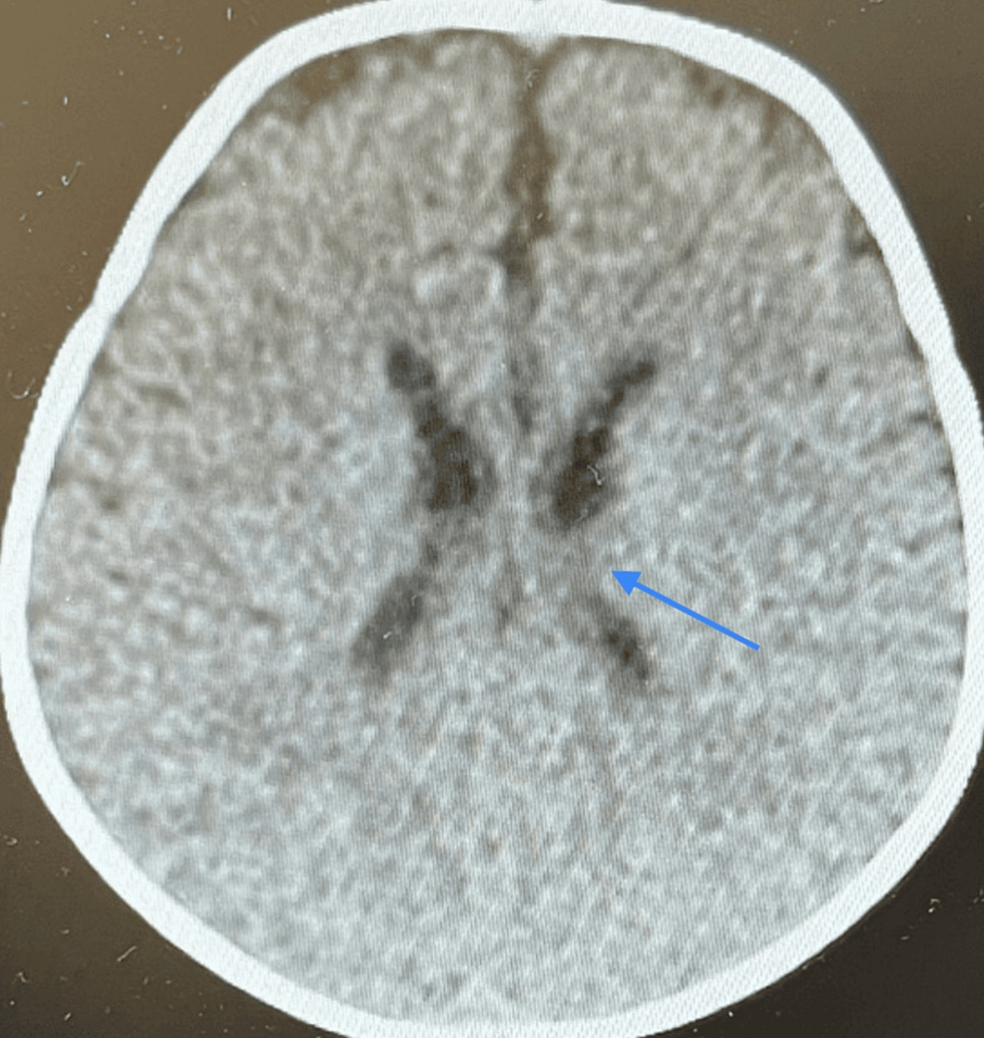
Non-contrast CT brain scan: thin corpus callosum.

**Figure 12:**
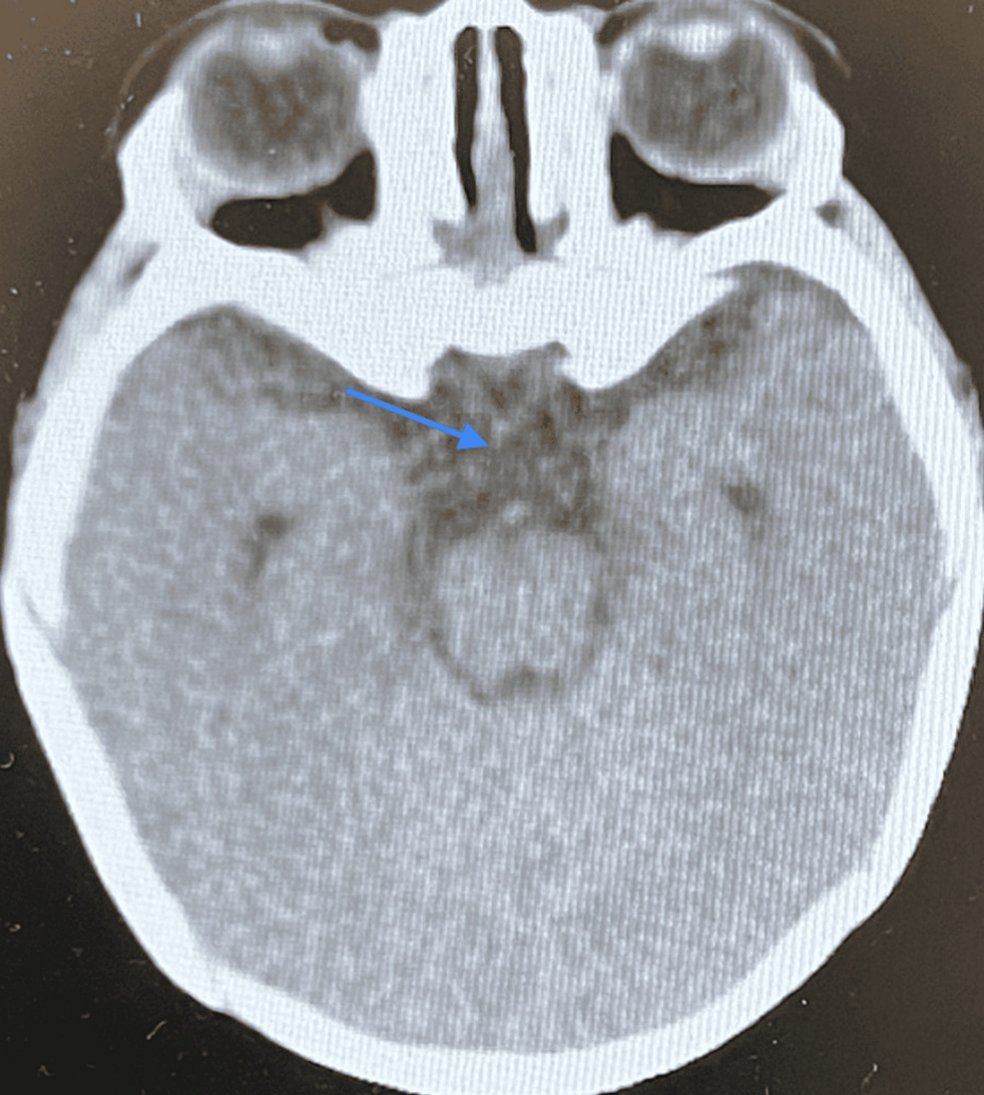
Non-contrast CT brain scan: posterior fossa structures degraded.

**Figure 13:**
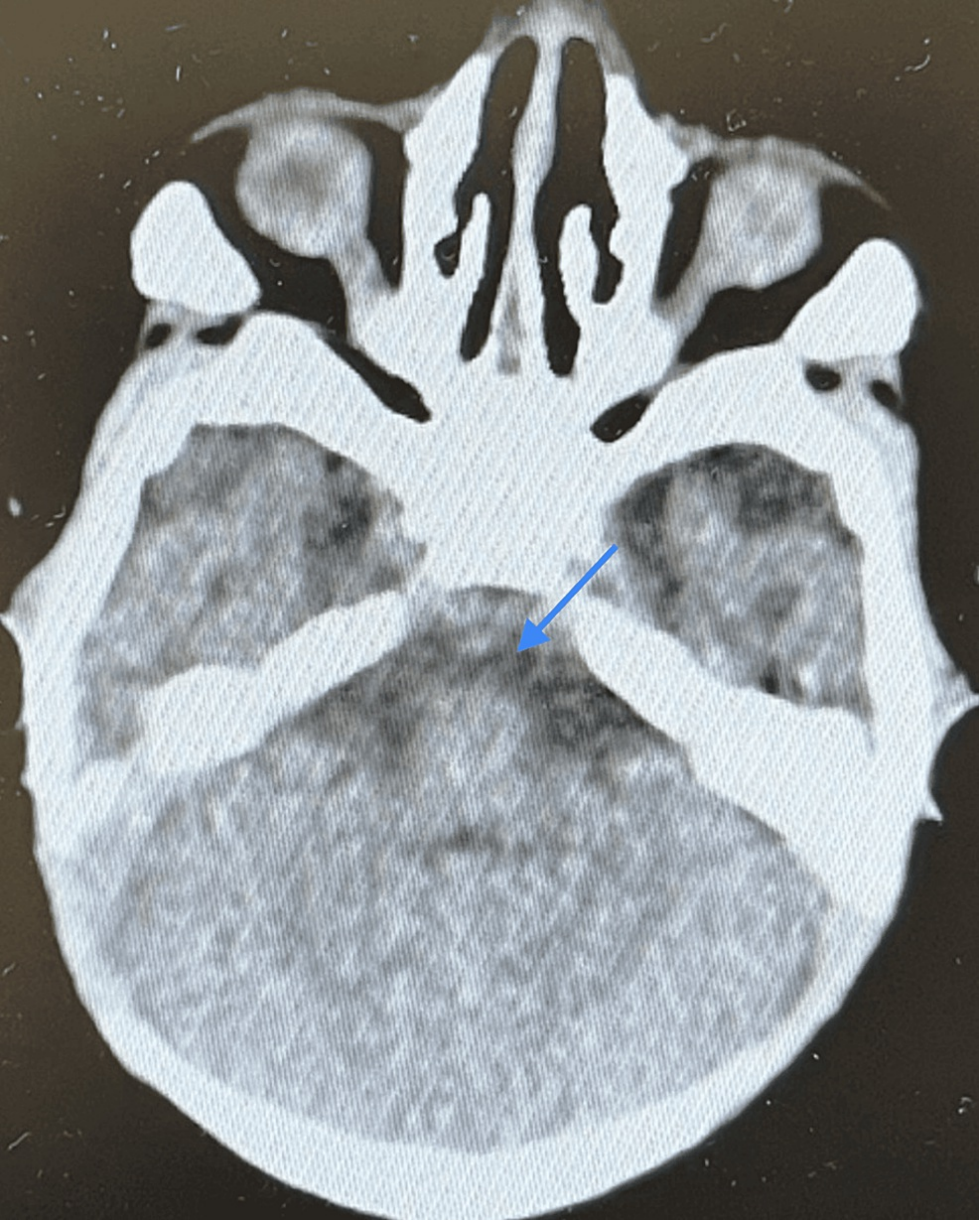
Non-contrast CT brain: posterior fossa structures degraded.

A non-contrast CT brain scan showed a thin corpus callosum (Figures 10-11), no intracranial hemorrhage or infarctions, and gray-white matter differentiation was maintained with no midline shifts. Ventricles, sulci, and basal cisterns were normal. The posterior fossa structures were degraded by beam-hardening artifacts (Figures 12-13). However, they appeared grossly unremarkable. Opacification of the pneumatized ethmoid air cells and bilateral maxillary sinuses was present, but bilateral opacification of the mastoid air cells was more on the left side. Visualized osseous structures appeared unremarkable, and no fracture line was apparent.

The echocardiogram revealed normal cardiac segments (SDS) with the following findings: the atria were normal, a small patent foramen ovale (PFO) was noted, and both the mitral and tricuspid valves appeared normal. Additionally, the aortic and pulmonary valves were within normal limits. Both the right and left ventricles exhibited normal morphology, and the ventricular septum was intact. However, there was evidence of branch stenosis in the pulmonary arteries, with a peak gradient of 28 mmHg. The aortic arch appeared normal, with no evidence of patent ductus arteriosus (PDA) or coarctation. Cardiac function was assessed as normal. Overall, the echocardiogram findings indicated branch pulmonary artery stenosis, which typically resolves spontaneously within a few months.

The pregnancy history was reviewed with an obstetrician, and all screenings during pregnancy showed negative results for syphilis. Hence, the disease could have been transmitted to the baby while delivering from the vagina during labor as the mother is presumed to be in the primary stage of the disease. The baby received a 14-day course of penicillin G through a peripherally inserted central catheter (PICC) line. Long-term follow-up with various specialists was recommended, including ophthalmology, orthopedics, cardiology, and audiology.

Furthermore, the parents were tested for various infections, specifically toxoplasmosis, HIV, CMV, hepatitis profile, Treponema Pallidum Hemagglutination Assay (TPHA), VDRL, and HSV and they needed to receive treatment accordingly and follow up with an adult infectious disease doctor.

## Discussion

The WHO emphasizes that congenital syphilis stands as the most preventable cause of stillbirth and advocates for antenatal screening programs for all pregnant women, alongside treatment of all positive cases and their partners [5-7]. In our patient's case, her mother underwent syphilis screening during a routine visit, and TORCH results returned negative. Clinical symptoms in our patient manifested at 2 months. This study shows the majority of infants with congenital syphilis are asymptomatic at birth and in most untreated children clinical features manifest within the first 3 months of life (Herremans T, Kortbeek L, Notermans DW. A review of diagnostic tests for congenital syphilis in newborns. Eur J Clin Microbiol Infect Dis. 2010;29(5):495) [7].

Infants with congenital syphilis typically present initially with classic symptoms such as maculopapular rash on the face, hands, and feet, along with failure to thrive and hepatosplenomegaly [1,8-10]. However, in our study, the infant presented at the hospital with the chief complaint of poor weight gain, leading to delayed vaccination. Upon physical examination, uncommon findings were noted, including hypoactive and paralyzed right arm with swelling, which correlated with findings from one of the six studies reviewed [8]. Additionally, severe anemia was observed during the hospital course, necessitating a blood transfusion. Only one study from Seoul, Korea, reported a moderate degree of anemia in 50 infants with congenital syphilis [11]. Moreover, our case revealed severe abdominal distention and osteomyelitis, which were not documented in any of the six studies, although hepatosplenomegaly was consistent across all studies. Although rash was a consistent finding in the six studies reviewed [1,3,8-11], it was absent in our patient. These atypical and rare findings of congenital syphilis in our patient prompted pediatricians from various specialties to convene and investigate the underlying cause, as failure to diagnose congenital syphilis could lead to fatal outcomes [8].

To prevent the oversight of congenital syphilis during pregnancy, as occurred in our case, several studies have demonstrated that identifying syphilis infection during pregnancy and administering appropriate treatment can reduce the incidence of congenital syphilis by 97% [12]. Based on the patient's presentation and birth history, it is likely that the infant contracted the infection during contact with an infected vagina. The opportunity was identified where healthcare workers could have intervened to prevent and identify similar conditions [12]. The CDC recommends documenting the syphilis serological state of the mother at least once during pregnancy and at delivery before discharging the neonate [12]. Healthcare workers following this recommendation can increase the chances of detecting congenital syphilis missed during perinatal care.

## Conclusions

Congenital syphilis in children does not always exhibit typical symptoms; it can manifest with unusual presentations such as paralysis of the upper or lower limbs, anemia, and osteomyelitis, as observed in our case. Therefore, early screening during pregnancy and at delivery remains crucial to mitigate its incidence. This case highlights the diverse clinical presentations seen in congenital syphilis and emphasizes the importance of heightened awareness among healthcare providers to facilitate timely diagnosis and intervention, thereby averting severe complications and mortality in affected newborns.
